# ﻿First male description of *Urodeta longa* Sruoga & Kaila, 2019 from Thailand with identification keys to Asian species of *Urodeta* Stainton, 1869 (Lepidoptera, Elachistidae, Elachistinae)

**DOI:** 10.3897/zookeys.1250.157014

**Published:** 2025-08-26

**Authors:** Virginijus Sruoga, Lauri Kaila, Erkka Laine

**Affiliations:** 1 Institute of Biosciences, Life Sciences Center, Vilnius University, Saulėtekio Ave. 7, LT-10257 Vilnius, Lithuania Vilnius University Vilnius Lithuania; 2 Zoology Unit, Finnish Museum of Natural History, P.O. Box 17, FI-00014 University of Helsinki, Helsinki, Finland University of Helsinki Helsinki Finland; 3 Ecology and Genetics Research Unit, P.O. Box 3000, 90014 University of Oulu, Oulu, Finland University of Oulu Oulu Finland

**Keywords:** Asia, high barcode divergence, microlepidoptera, mining moths, morphology, taxonomy

## Abstract

A male of the little-known species *Urodeta longa* Sruoga & Kaila, 2019 is described for the first time based on material collected in northern Thailand. The species is diagnosed based on characters found in the habitus and genitalia, which are illustrated in detail. Conspecificity of male and female specimens is confirmed by DNA barcodes. Identification keys to all known Asian species of the genus *Urodeta* Stainton, 1869, based on male and female genitalia, are provided. Exceptionally high intra-generic barcode divergence among *Urodeta* species is reported.

## ﻿Introduction

*Urodeta* Stainton, 1869 is a small genus in the subfamily Elachistinae Bruand (Gelechioidea, Elachistidae), comprising 28 named (Sruoga 2024) and one described but unnamed species (Kaila 2011). The larvae are leaf miners in dicotyledonous plants in the families Cistaceae and Combretaceae (Sruoga and De Prins 2022); however, host plants are known for only one Mediterranean species, *U. hibernella* (Staudinger, 1859) (Stainton 1869) and an unnamed Australian species (Kaila 2011).

The moths are generally small to very small, with a wingspan ranging from 4.0 to 9.5 mm. The labial palpi are tiny, and the antennae are shorter than the forewings and relatively broad, particularly in males. The forewings typically have a dull grey or brownish pattern, sometimes with inconspicuous markings. The hindwings are narrow to very narrow. All currently known Asian species of *Urodeta* are superficially similar. The moths are small and pale-coloured with a somewhat maculate forewing pattern and indistinct wing markings. Species-level diagnoses are based exclusively on characters of the genitalia.

In the male genitalia, this genus is characterized by having anteriorly directed spines of the gnathos, the phallus distally fused with the ventral shield of the juxta, and the absence of a digitate process. In the female genitalia, the apophyses anteriores extend from the middle of segment 8 and the apex is directed laterally in many species (Sruoga and De Prins 2011).

The known geographic range of the genus has been expanded significantly based on studies over the past 17 years. Once known only from the Mediterranean region, species of *Urodeta* are now known to occur in Asia (Sruoga and De Prins 2013; Sruoga and Rocienė 2018; Sruoga et al. 2019), sub-Saharan Africa (Mey 2007; Sruoga and De Prins 2009, 2011, 2022), Australia (Kaila 2011), and Central America (Sruoga 2024). Despite this wide distribution, the number of species documented on different continents and the scope of their study remain particularly limited. This pattern is attributable to the small size and dull colouration, as well as a poorly known life history.

Southeast Asia is a significant yet largely undersampled region in terms of microlepidoptera. The true diversity of Elachistinae, for instance, is notably neglected, particularly in Thailand. Ten species, all new to Thailand, with eight of them described as new were recently reported (Sruoga et al. 2019). Among these species, one described from a single female specimen belongs to the genus *Urodeta* (*U. longa* Sruoga & Kaila, 2019), which is currently the only known species of this genus in Thailand. Our research, based on newly obtained material, reveals the male of *U. longa*, described and illustrated here for the first time. Conspecificity of male and female specimens is confirmed by DNA barcoding.

## ﻿Materials and methods

### ﻿Dissection and photographic documentation

Adult specimens were examined externally using a Nikon SMZ445 stereomicroscope and measured using an ocular micrometer. The forewing length was measured along the costa, from the wing base to the apex of the terminal fringe scales, and the head width was measured between the inner edges of the antennal bases. Genitalia were prepared following the standard method described by Robinson (1976), adapted for the Elachistinae (Traugott-Olsen and Nielsen 1977). The genitalia were studied, and some morphological structures were photographed in glycerol before permanent slide-mounting in Euparal. The male genital capsule was stained with fuchsin, the abdominal pelt, and the female genitalia with chlorazol black E (Direct Black 38/Azo Black). The genital morphology was examined using a Leica DM6 B microscope. Photographs of adults were taken using a Canon EOS 80D camera fitted with an MP-E 65 mm Canon macro lens mounted on a macro rail (MJKZZ Qool Rail). Genitalia photographs were taken with a Leica DM6 B microscope and a Leica K3C digital camera. Zerene Stacker 1.0, with a retouch function, was used for image stacking. All images were optimized and arranged into plates using Adobe Photoshop CC 2019.

### ﻿Terminology and depository

The descriptive terminology of morphological structures follows Traugott-Olsen and Nielsen (1977) and Kaila (1999, 2011). The material used in this paper is deposited in the collection of the Finnish Museum of Natural History, Helsinki, Finland (**MZH**).

### ﻿Molecular analysis

DNA Barcode Gap Analysis: the sequence used was the standard DNA barcode, i.e., cytochrome oxidase subunit 1 5′ region (CO1-5P). In total, the data set comprises 21 *Urodeta* records, with 21 sequences meeting the requirements applied here. Nine of these represent *Urodeta longa*, one *U. inusta* Kaila, and the remaining 11 *Urodeta hibernella*. The name *Urodeta cisticolella* Stainton, a synonym of *U. hibernella*, is used for two of these records, following the information available on the BOLD Systems Database. The DNA barcode distance analysis was performed using all public *Urodeta* data on BOLD, as of 15 March 2025. For alignment of sequences BOLD aligner (Amino Acid based HMM) was used. The pairwise distance (*p*-distance) model for nucleotide substitution was used as the model for measuring genetic divergences. Only sequences with at least 400 base pairs were used. Records containing stop codons, misidentification, or other error-flags as well as contaminated records were excluded from the analysis. Ambiguous bases were handled with Pairwise Deletion. The distances for nearest neighbors were measured using a species pool of three *Urodeta* species. Information on genetic distances within and among species was assembled in a distance matrix (Suppl. material 1). Further details of sequenced material including complete voucher data and images of specimens can be accessed in the public dataset DS-URODLONG “*Urodeta* species” https://doi.org/10.5883/DS-URODLONG.

## ﻿Results

Maximum intraspecific barcode difference (*n* = 9) in *Urodeta longa* is 1.69%, and *U. hibernella* 2.16%. Of *U. inusta*, only one barcode is available. Maximum interspecific distances are 13.8% between *U. inusta* and *U. longa*, 14.93% *U. hibernella* and *U. longa*, and 18.62% between *U. inusta* and *U. hibernella* (See Suppl. material 1).

### ﻿Key to the Asian species of the *Urodeta* Stainton, based on male genitalia (male of *U. pectena* Sruoga & Rocienė) is unknown and not included in the key)

**Table:** 

1	Valva divided into two separate lobes: the ventral lobe is almost parallel-sided and rounded apically, while the dorsal lobe is triangular (Sruoga and De Prins 2013: figs 7, 8)	***U. noreikai* Sruoga & De Prins**
–	Valva not divided into two separate lobes	**2**
2	Spine where sacculus meets cucullus is shorter than its width at the base (Sruoga and Rocienė 2018: figs 9–11)	***U. jurateae* Sruoga & Rocienė**
–	Spine where sacculus meets cucullus is longer than its width at the base (Figs 3A, D, G, H, 4A–C)	***U. longa* Sruoga & Kaila**

### ﻿Key to the Asian species of the *Urodeta* Stainton, based on female genitalia

**Table:** 

1	Corpus bursae without signum	**2**
–	Corpus bursae with signum	**3**
2	Antrum large, oval, strongly sclerotized, with about 21–27 large and several much smaller internal spines (Sruoga et al. 2019: figs 3, 6; this paper Fig. 4G–I)	***U. longa* Sruoga & Kaila**
–	Antrum without internal spines; dorsal wall with large, strongly sclerotized paired plate (Sruoga and Rocienė 2018: figs 14, 15)	***U. jurateae* Sruoga & Rocienė**
3	Signum rounded, dentate, surrounded by spines arranged radially (Sruoga and De Prins 2013: figs 13, 16, 17)	***U. noreikai* Sruoga & De Prins**
–	Signum comb-shaped, formed from nine stout teeth, that vary in size (Sruoga and Rocienė 2018: fig. 19)	***U. pectena* Sruoga & Rocienė**

#### ﻿ Urodeta longa

Taxon classificationAnimaliaLepidopteraElachistidae

Sruoga & Kaila, 2019

F4E4014D-F5B3-5CF7-9331-74FD3082C1B6

Figs 1 , 2 , 3 , 4

##### Material examined.

Thailand • 1 ♀ (holotype); Lampang, Muban Phichai; 4 Mar. 2017; gen. prep. VS1/29.03.19; http://id/luomus.fi/KR.36156 [barcode unsuccessful] • 1 ♂; Lampang, Chae Hom; 18°43.488'N, 99°40.614'E; 02 Mar. 2019; gen. prep. VS603 • 1 ♂; Thailand, Lampang, Chae Hom; 340 m a.s.l.; 18°43.3166'N, 99°33.1833'E; 10 Dec. 2020; http://id/luomus.fi/F.469302; • 1 ♀; Thailand, Lampang, Muban Phichai; 240 m a.s.l.; 18°18.25'N, 99°31.1'E; 17 Feb. 2020; http://id/luomus.fi/F.470851; gen. prep. VS611 • 1 ♂; same locality; 19 Feb. 2020; http://id/luomus.fi/F.470853; gen. prep. VS610 • 2 ♂; same locality; 20 Feb. 2020; http://id/luomus.fi/F.470854; gen. prep. VS638; http://id/luomus.fi/F.470855; gen. prep. VS613 • 1 ♀; same locality; 23 Feb. 2020; http://id/luomus.fi/F.470856 • 1 ♂; same locality; 06 Jan. 2021; http://id/luomus.fi/F.469303; gen. prep. VS612 • 2 ♂; same locality; 03 Mar. 2021; http://id/luomus.fi/F.469307; gen. prep. VS639; http://id/luomus.fi/F.469300; gen. prep. VS604 • 2 ♀; same locality; 06 Mar. 2021; http://id/luomus.fi/F.469305; gen. prep. VS614; http://id/luomus.fi/F.469306; M.J. Pellinen leg.; MZH.

##### Diagnosis.

*Urodeta longa* is mostly similar to *U. jurateae* with both having a valva not divided into two separate lobes, a strongly sclerotization in the caudal part of the female genitalia, and the absence of signa. The main differences between *U. longa* and *U. jurateae* are: (1) the spine where sacculus meets cucullus is longer than its width at the base in *U. longa*, whereas in *U. jurateae* it is shorter than its width at the base; (2) the phallus in *U. longa* is without dorsal carina, in *U. jurateae* phallus is with paired palmate dorsal carina; (3) the antrum in *U. longa* is strongly sclerotized, with about 21–27 large and several small internal spines and dorsal wall without sclerotized plate, whereas in *U. jurateae* the antrum is membranous, with minute internal spines and the dorsal wall with large strongly sclerotized paired plate.

**Figure 1.**
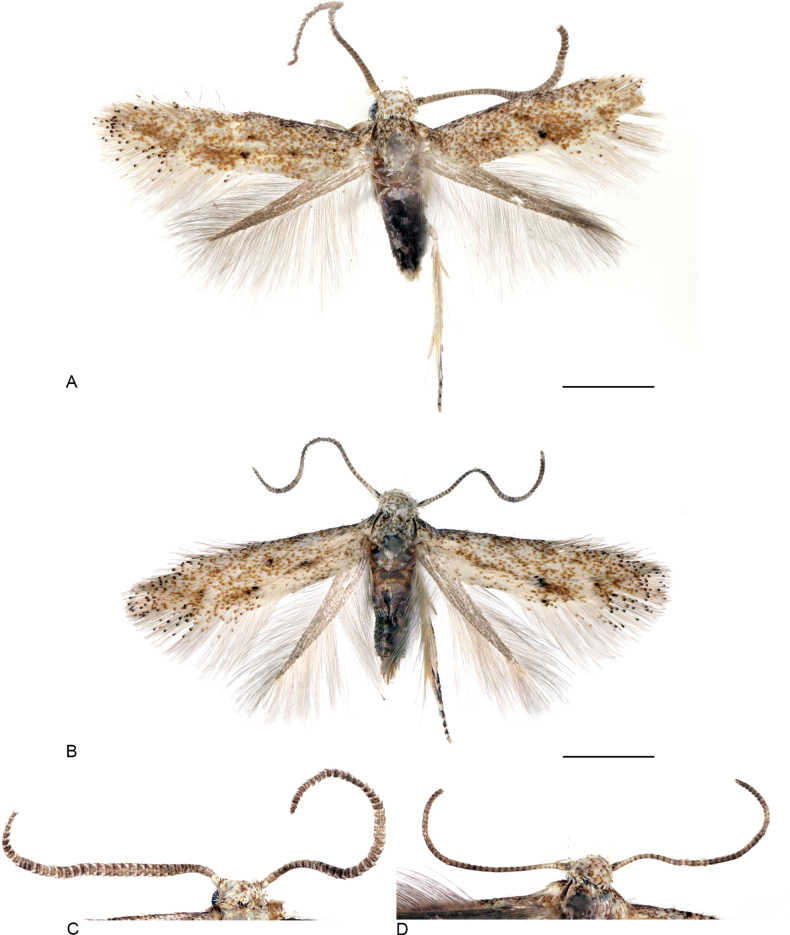
Adults of *Urodeta longa.* A. Male, specimen F.470854; B. Female, specimen F.469305; C. Male head and antennae, specimen F.469302; D. Female head and antennae, specimen F.469306. Scale bars: 1 mm.

##### Description of male.

Forewing length 2.5–3.4 mm; wingspan 5.5–7.2 mm (*n* = 8). Head: frons, vertex, and neck tuft white, weakly mottled with light brown tipped scales; labial palpus straight, 0.5 as long as the width of head, white, with very weak admixture of pale brown; antenna 0.8 as long as length of forewing, scape white, mottled with light brown tipped scales, pecten white; flagellum brownish grey, weakly annulated with paler rings in basal part, flagellomeres bowl-shaped, unusually wide, especially in central part of flagellum. Thorax and tegula strongly mottled with scales basally white and distally greyish brown; forewing strongly mottled with scales basally white and distally from creamy brown to greyish brown; black, brown-tipped scales forming two small, blurred spots transversally arranged just before middle of wing, another small black-brown spot on fold at 1/4 from base of wing; fringe scales creamy white with some blackish brown tipped scales. Hindwing brownish grey, its fringe scales somewhat paler.

**Figure 2.**
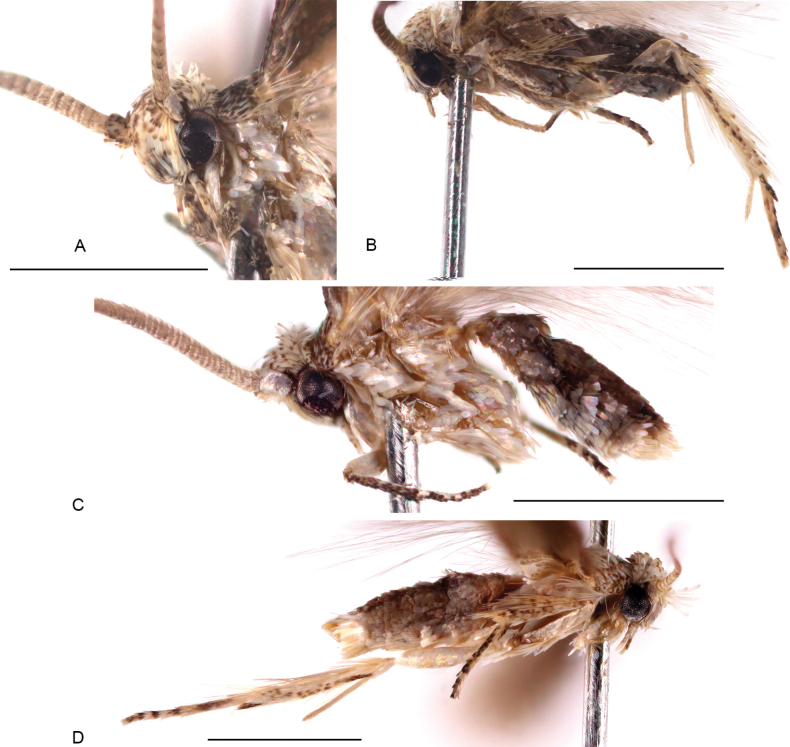
Adults of *Urodeta longa.* A. Male head, fronto-lateral view, specimen F.469307; B. Male, lateral view, specimen F.469302; C. Ditto, specimen F.469307; D. Female, lateral view, specimen F.469306. Scale bars: 1 mm.

***Male genitalia*.** Tegumen weakly sclerotized, lateral margin strongly folded inwards. Uncus approximately 0.5× as long as tegumen, posterior margin membranous, without paired lobes. Basal arms of gnathos narrow, basally strongly sclerotized and membranous distally, spinose knob of gnathos as large as length of uncus, slightly ovate, spines anteriorly directed. Valva short and broad; costa somewhat wrinkled, weakly sclerotized, covered with several short setae, distally with sharp curved tip; sacculus convex, distally with sharp, claw-shaped process before terminal emargination; cucullus narrow and elongate, apex with small upcurved spine; inner processes of valva fused apically, with a few thin setae, forming weakly sclerotized transtilla. Ventral shield of the juxta about 2× as long as wide with strongly sclerotized median ridge; lateral membranous extension of the juxta apically bilobed, with few short setae, partly surrounds the phallus; juxta lobes approximately 4/5 as long as of ventral shield of juxta, distally tapered, with a few tiny setae. Vinculum U-shaped, narrow. Phallus apically fused to the juxta, longer than valva, slightly dilated in the proximal part, gradually tapered towards pointed apex, which is s-shaped in lateral view; insertion of ductus ejaculatorius dorso-laterally directed; caecum small; vesica with 6 or 7 large, straight cornuti, 4–6 smaller, bent ones in a cluster and about 10–15 small cornuti of varying size.

**Figure 3.**
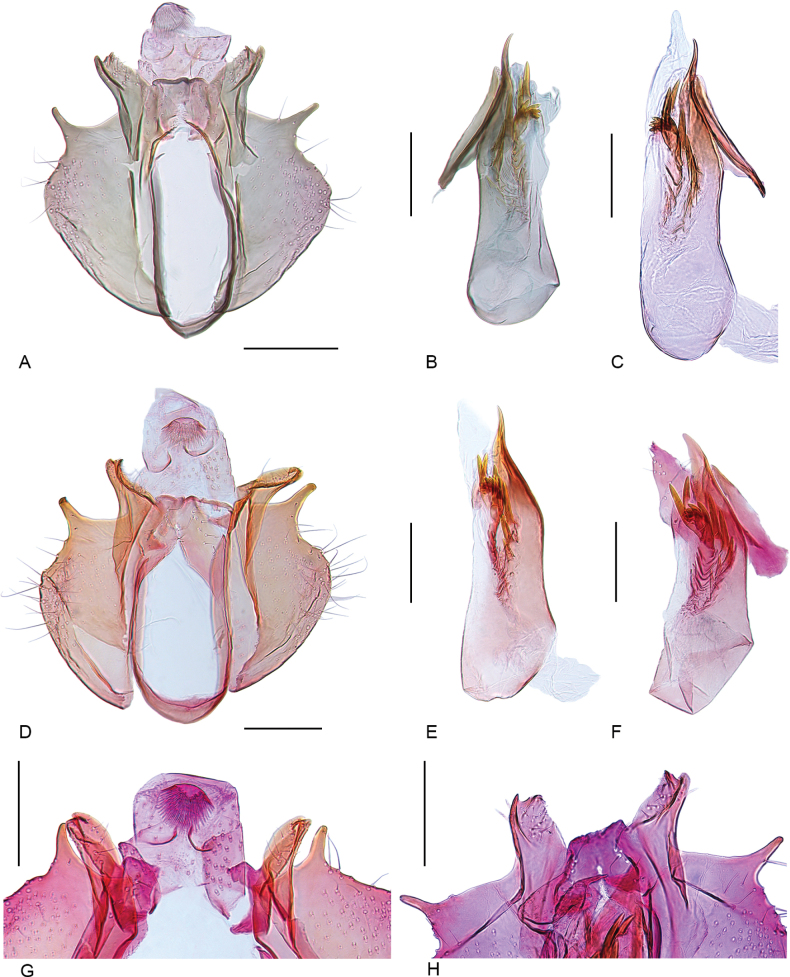
Male genitalia of *Urodeta longa.* A. General view, phallus removed, slide no. VS610; B. Phallus, slide no. VS610; C. Phallus, slide no. VS604; D. General view, phallus removed, slide no. VS612; E. Phallus, slide no. VS612; F. Phallus, slide no. VS613; G. Apical part of genitalia, slide no. VS613; H. Apical part of genitalia, slide no. VS603. Scale bars: 0.1 mm.

##### Biology.

The host plant and early stages are unknown. Adults fly from mid-December until early March.

##### Distribution.

Known only from northern Thailand.

##### Barcode information.

Nine specimens of *U. longa* were successfully barcoded. Intraspecific variation was 1.69%, branch length/distance to closest intra-generic match, *U. hibernella* (with the synonymic name *U. cisticolella* (LNAUW3357-18), was 13.08%. See Discussion below.

**Figure 4.**
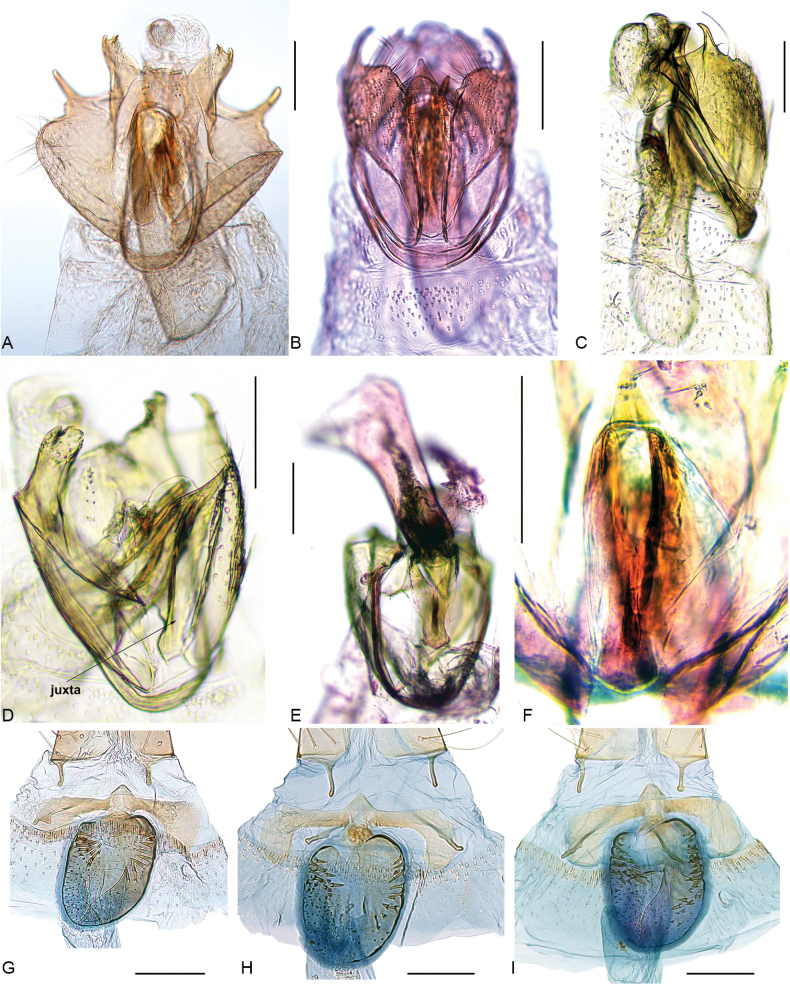
Genitalia of *Urodeta longa.* A. Male genitalia, ventral view, specimen F.470854; B. Male genitalia, ventral view, valvae not spread, specimen F.470853; C. Male genitalia, lateral view, specimen F.469300; D. Male genitalia, latero-ventral view, specimen: Thailand, Lampang, Chae Hom, 18°43.488'N, 99°40.614'E, 02.iii.2019; E. Male genitalia, dorsal view, phallus lifted upwards, specimen F.469303; F. Male genitalia, juxta, specimen F.470854; G. Female genitalia, antrum area, holotype; H. Ditto, slide no. VS611; I. Ditto, slide no. VS614 (A–F in glycerol before permanent mounting in Euparal). Scale bars: 0.1 mm.

## ﻿Discussion

*Urodeta longa* was previously known from a single, abraded female specimen collected in northern Thailand (Sruoga et al. 2019). Here, we present additional illustrations of an unabraded female (Fig. 1B) derived from newly obtained material, thereby complementing the original description.

Superficially, the wing pattern of males closely resembles that of females, although males are slightly larger. The most pronounced sexual dimorphism is evident in the shape of the antennae. Female antennae are similar to those of most of the species within Elachistinae, whereas those of males are notably broader and flattened. Broad male antennae are characteristic of several other species of *Urodeta*, but the antennae of males of *U. longa* are among the broadest known in this genus (Fig. 1C).

The moths of the genus *Urodeta* are among the smallest within Elachistinae. Their male genitalia and some of their parts are often difficult to observe and interpret due to their small size, weak sclerotization, or challenges in obtaining high-quality slides. One such subtle structure is the juxta, which in *Urodeta* is tightly fused to the distal part of the phallus. Consequently, during slide preparation, when removing the phallus from the genital capsule for better visibility, a part or all of the juxta may be torn. Therefore, it is advisable to document the shape of the juxta in glycerol as clearly as possible before removing the phallus from the genital capsule and embedding it in Euparal.

The shape of the juxta, with its tapering lobes, in *U. longa* is most clearly visible in dorsal view, with the phallus lifted upwards (Fig. 4E). The strongly sclerotized ventral shield of the juxta is practically invisible if the phallus is not removed from the genital capsule, or if the juxta is partially torn and remains attached to the distal part of the phallus (Fig. 3B, C, F).

Another distinct diagnostic character is the claw-shaped spine on the distal part of the sacculus. Its exact shape is difficult to interpret on the slide, as its curvature causes the apex to appear rounded (Figs 3A, D, G, H, 4A). However, when viewed from the lateral side, it is clearly pointed (Fig. 4C). The same applies to the cucullus, the shape of which can appear different depending on whether the preparation is compressed less (Fig. 3G) or more (Fig. 3H) when mounted on a permanent slide. The cornuti illustrated in Fig. 3B, F represent extremities, while some of the other display intermediates. Therefore, we consider all these specimens conspecific, as also supported by DNA barcodes.

The morphology of the male genitalia is taxonomically often more informative than the female genitalia in Elachistinae. However, in the case of *U. longa*, the morphology of the female genitalia is quite informative, particularly in the features of the antrum with its complex set of spines. The examination of additional material revealed some variation in the number and size of spines on the antrum (Fig. 4G–I), as well as in the size of swellings on the apophysis anterioris, which range from large (Fig. 4G) to rather small (Fig. 4H, I).

Unfortunately, nothing is known about the immature stages and host plants of *U. longa*. Much more research is needed to better understand this and other Asian species of *Urodeta*.

Interspecific variability among the three *Urodeta* species with complete barcodes is high compared to other apoditrysian Lepidoptera, up to 18.62% (Suppl. material 1). These species would not be among the closest matches to each other in the BOLD identification engine. While the closest matches of any of these species belong to Lepidoptera, they are nearly randomly distributed within the order, including, for instance, representatives of Papilionoidea and Noctuoidea, instead of congeners. The overall magnitude of divergence is not, however, quite unique in Lepidoptera. Lee and Roh (2025) reported intra-generic divergences of same magnitude in the basal ditrysian lepidopteran genus *Eudarcia* Clemens (Meessiidae in the tineoid family assemblage). Other genes of *Urodeta* are also quite divergent as compared to other Gelechioidea, which may explain the instability of the position of the genus within the superfamily when solely based on genomic data (Heikkilä et al. 2014). Morphology nevertheless suggests a close relationship of *Urodeta* with other elachistine genera (Kaila and Sugisima 2011; De Prins and Sruoga 2012). The intrageneric barcode divergences in *Urodeta* are, to our knowledge, the largest reported in Apoditrysia. Pentinsaari et al. (2014) reported the average mean nearest neighbor divergences in Lepidoptera, including all hierarchical levels, to be 5.80%, about a third of that within *Urodeta*.

## Supplementary Material

XML Treatment for Urodeta longa

## ﻿Additional information

### Use of AI

No use of AI was reported.

### Author ORCIDs

Virginijus Sruoga https://orcid.org/0000-0002-5687-8299

Lauri Kaila https://orcid.org/0000-0003-0277-1872

Erkka Laine https://orcid.org/0009-0000-4881-2110

### Data availability

All of the data that support the findings of this study are available in the main text.

## Supplementary materials

Supplementary material 1 Pairwise genetic distance matrix of COI sequences among *Urodeta* species Data type pdf https://binary.pensoft.net/file/1402143This dataset is made available under the Open Database License (http://opendatacommons.org/licenses/odbl/1.0/). The Open Database License (ODbL) is a license agreement intended to allow users to freely share, modify, and use this Dataset while maintaining this same freedom for others, provided that the original source and author(s) are credited.Virginijus Sruoga, Lauri Kaila, Erkka Laine

## References

[B1] De Prins J Sruoga V (2012) A review of the taxonomic history and biodiversity of the genus *Urodeta* (Lepidoptera: Elachistidae: Elachistinae), with description of new species. Zootaxa 3488(1): 41–62. 10.11646/zootaxa.3488.1.2

[B2] Heikkilä M Mutanen M Kekkonen M Kaila L (2014) Morphology reinforces proposed molecular phylogenetic affinities: A revised classification for Gelechioidea (Lepidoptera). Cladistics 30(6): 563–589. 10.1111/cla.12064 34794251

[B3] Kaila L (1999) Phylogeny and classification of the Elachistidae*s.s.* (Lepidoptera: Gelechioidea). Systematic Entomology 24(2): 139–169. 10.1046/j.1365-3113.1999.00069.x

[B4] Kaila L (2011) Elachistine moths of Australia (Lepidoptera: Gelechioidea: Elachistidae). Monographs on Australian Lepidoptera (Vol. 11). CSIRO Publishing, Melbourne, x + 443 pp. 10.1071/9780643103481

[B5] Kaila L Sugisima K (2011) 1. Phylogeny, subfamily definition and generic classification. In: Kaila L (Ed.) Elachistine moths of Australia (Lepidoptera: Gelechioidea: Elachistidae).Monographs on Australian Lepidoptera (Vol. 11). CSIRO Publishing, Melbourne, 7–22. 10.1071/9780643103481

[B6] Lee D-J Roh SJ (2025) A new species of the genus *Unilepidotricha* (Lepidoptera, Meessiidae) from Ulleungdo island, Korea. Zootaxa 5613(3): 585–592. 10.11646/zootaxa.5613.3.10 40173486

[B7] Mey W (2007) Microlepidoptera: Smaller families. In: Mey W (Ed.) The Lepidoptera of the Brandberg Massif in Namibia. Part 2.Esperiana Memoir 4: 9–30.

[B8] Pentinsaari M Hebert PDN Mutanen M (2014) Barcoding beetles: A regional survey of 1872 species reveals high identification success and unusually deep interspecific divergences. PLoS ONE 9(9): e108651. 10.1371/journal.pone.0108651 PMC4177932 25255319

[B9] Robinson GS (1976) The preparation of slides of Lepidoptera genitalia with special reference to the microlepidoptera. Entomologist’s Gazette 27: 127–132.

[B10] Sruoga V (2024) The first Neotropical record of the genus *Urodeta* (Lepidoptera: Elachistidae: Elachistinae) with keys to the world species and a description of a new species from Honduras. Insects 15(12): e941. 10.3390/insects15120941 PMC11677709 39769542

[B11] Sruoga V De Prins J (2009) The Elachistinae (Lepidoptera: Elachistidae) of Kenya with descriptions of eight new species. Zootaxa 2172(1): 1–31. 10.11646/zootaxa.2172.1.1

[B12] Sruoga V De Prins J (2011) New species of Elachistinae (Lepidoptera: Elachistidae) from Cameroon and the Democratic Republic of the Congo. Zootaxa 3008(1): 1–32. 10.11646/zootaxa.3008.1.1

[B13] Sruoga V De Prins J (2013) A new species of *Urodeta* (Lepidoptera: Elachistidae: Elachistinae) from Nepal, the first record of the genus from Asia, showing an ancient distribution pattern. Zootaxa 3599(1): 94–100. 10.11646/zootaxa.3599.1.9 24583819

[B14] Sruoga V De Prins J (2022) New species of *Urodeta* Stainton, 1869 (Lepidoptera, Elachistidae, Elachistinae) from Ghana and Democratic Republic of the Congo, with identification keys to the Afrotropical species of the genus. ZooKeys 1089: 25–36. 10.3897/zookeys.1089.79716 35586601 PMC8933387

[B15] Sruoga V Rocienė A (2018) Three new species of Elachistidae (Lepidoptera: Gelechioidea) from India. Zootaxa 4394(4): 575–585. 10.11646/zootaxa.4394.4.8 29690351

[B16] Sruoga V Kaila L Rocienė A (2019) The Elachistinae (Lepidoptera: Gelechioidea, Elachistidae) of Thailand, with description of eight new species. European Journal of Taxonomy 574(574): 1–34. 10.5852/ejt.2019.574

[B17] Stainton HT (1869) The Tineina of Southern Europe. John van Voorst, London, viii +370 pp. 10.5962/bhl.title.26568

[B18] Traugott-Olsen E Nielsen ES (1977) The Elachistidae (Lepidoptera) of Fennoscandia and Denmark. Fauna Entomologica Scandinavica 6: 1–299. 10.1163/9789004273290

