# Supplementary material for: ﻿First male description of Urodeta longa Sruoga & Kaila, 2019 from Thailand with identification keys to Asian species of Urodeta Stainton, 1869 (Lepidoptera, Elachistidae, Elachistinae)
Source: Zookeys. 2025 Aug 26;1250:1–12. doi: 10.3897/zookeys.1250.157014 (PMC12406021; doi:10.3897/zookeys.1250.157014)
Supplement: Supplementary material 1 — Pairwise genetic distance matrix of COI sequences among Urodeta species [file zookeys-1250-001_article-157014__-s001.pdf]

Table S1. Pairwise genetic distance matrix of COI sequences among *Urodeta* species. Numbers from 1 to 21 are given for each record in the dataset. Process ID codes are provided as a reference. Values are given as percentages. The sequences were aligned with the BOLD Amino Acid Aligner and pairwise deletion was used. All sequences included are above 400 basepairs in length.

Species	1	2	3	4	5	6	7	8	9	10	11	12	13	14	15	16	17	18	19	20	21
1 *Urodeta inusta* ANICH472-10	-																				
2 *Urodeta longa* LEFIJ33123-23	14.18	-																			
3 *Urodeta longa* LEFIJ33125-23	14.00	0.15	-																		
4 *Urodeta longa* LEFIJ33126-23	14.55	0.31	0.46	-																	
5 *Urodeta longa* LEFIJ33128-23	14.00	0.15	0.00	0.46	-																
6 *Urodeta longa* LEFIJ33133-23	14.36	0.15	0.31	0.15	0.31	-															
7 *Urodeta longa* LEFIJ33134-23	14.18	0.31	0.46	0.31	0.46	0.46	-														
8 *Urodeta longa* LEFIJ33136-23	14.18	0.46	0.61	0.46	0.61	0.61	0.46	-													
9 *Urodeta longa* LEFIJ33137-23	13.81	1.38	1.54	1.69	1.54	1.54	1.38	1.54	-												
10 *Urodeta longa* LEFIJ33138-23	14.36	0.15	0.31	0.15	0.31	0.31	0.15	0.31	1.54	-											
11 *Urodeta hibernella* LEFIH047-10	17.77	15.67	15.51	15.82	15.51	15.82	15.67	15.67	16.44	15.67	-										
12 *Urodeta hibernella* LNAUV1252-17	17.96	15.29	15.11	15.47	15.11	15.47	15.29	15.29	15.11	15.29	1.25	-									
13 *Urodeta hibernella* LNAUV1253-17	17.74	15.11	14.93	15.29	14.93	15.29	15.11	15.11	14.93	15.11	1.43	0.18	-								
14 *Urodeta hibernella* LNAUV1254-17	17.96	15.29	15.11	15.47	15.11	15.47	15.29	15.29	15.11	15.29	1.25	0.00	0.18	-							
15 *Urodeta cisticolella* LNAUW3357-18	18.62	13.08	13.30	13.30	13.30	13.30	13.08	13.08	13.53	13.08	0.66	0.67	0.89	0.67	-						
16 *Urodeta cisticolella* LNAUW3358-18	18.34	13.75	13.53	13.97	13.53	13.97	13.75	13.75	13.75	13.75	0.44	0.00	0.22	0.00	0.66	-					
17 *Urodeta hibernella* LPSYA8183-23	18.32	15.21	15.36	15.36	15.36	15.36	15.21	15.21	15.67	15.21	1.53	1.61	1.79	1.61	1.10	0.88	-				
18 *Urodeta hibernella* LPSYA8184-23	18.23	15.21	15.05	15.36	15.05	15.36	15.21	15.21	15.67	15.21	1.54	1.98	2.16	1.98	1.11	0.89	1.69	-			
19 *Urodeta hibernella* PHLAD852-11	17.77	15.67	15.51	15.82	15.51	15.82	15.67	15.67	16.44	15.67	0.00	1.25	1.43	1.25	0.66	0.44	1.53	1.54	-		
20 *Urodeta hibernella* PHLAD853-11	17.58	15.51	15.36	15.67	15.36	15.67	15.51	15.51	16.28	15.51	0.15	1.07	1.25	1.07	0.44	0.22	1.38	1.38	0.15	-	
21 *Urodeta hibernella* GBGL41881-19	17.77	15.67	15.51	15.82	15.51	15.82	15.67	15.67	16.44	15.67	0.31	1.25	1.43	1.25	0.66	0.44	1.53	1.54	0.31	0.15	-
